# Aquatic urban ecology at the scale of a capital: community structure and interactions in street gutters

**DOI:** 10.1038/ismej.2017.166

**Published:** 2017-10-13

**Authors:** Vincent Hervé, Boris Leroy, Albert Da Silva Pires, Pascal Jean Lopez

**Affiliations:** 1 Laboratory of Microbiology, Institute of Biology, University of Neuchâtel, Neuchâtel, Switzerland; 2 Laboratory of Biogeosciences, Institute of Earth Surface Dynamics, University of Lausanne, Lausanne, Switzerland; 3 Unité Biologie des Organismes et Ecosystèmes Aquatiques (BOREA), Sorbonne Université, Centre National de la Recherche Scientifique (CNRS-7208), Muséum National d'Histoire Naturelle, Université Pierre et Marie Curie, Université de Caen Normandie, Institut de Recherche pour le Développement (IRD-207), Université des Antilles, Paris, France; 4 GEOCONCEPT, Bagneux, France

## Abstract

In most cities, streets are designed for collecting and transporting dirt, litter, debris, storm water and other wastes as a municipal sanitation system. Microbial mats can develop on street surfaces and form microbial communities that have never been described. Here, we performed the first molecular inventory of the street gutter-associated eukaryotes across the entire French capital of Paris and the non-potable waters sources. We found that the 5782 OTUs (operational taxonomic units) present in the street gutters which are dominated by diatoms (photoautotrophs), fungi (heterotrophs), Alveolata and Rhizaria, includes parasites, consumers of phototrophs and epibionts that may regulate the dynamics of gutter mat microbial communities. Network analyses demonstrated that street microbiome present many species restricted to gutters, and an overlapping composition between the water sources used for street cleaning (for example, intra-urban aquatic networks and the associated rivers) and the gutters. We propose that street gutters, which can cover a significant surface area of cities worldwide, potentially have important ecological roles in the remediation of pollutants or downstream wastewater treatments, might also be a niche for growth and dissemination of putative parasite and pathogens.

## Introduction

Biodiversity in urban areas is attracting more and more attention, with studies investigating the microbial diversity of various built environments such as offices (Chase *et al.*, 2016), the indoor air of shopping centers (Tringe *et al.*, 2008) and subways (Leung *et al.*, 2014), the surfaces of subway systems (Afshinnekoo *et al.*, 2015), urban parks and conservation areas (Ramirez *et al.*, 2014) or aquatic urban waters (Drury *et al.*, 2013; McLellan *et al.*, 2015). In these various intra-urban habitats, the biodiversity of protists (Genitsaris *et al.*, 2014; Grossmann *et al.*, 2016) has been less well explored compared with birds, insects, land plants or prokaryotes (Grimm *et al.*, 2008; Bahcall, 2015; Beninde *et al.*, 2015).

Among the protists, the unicellular photoautotrophic organisms attract a particular attention because they have various ecological roles, such as primary production and regulation of nutrients cycling, and because they can serve as indicators to assess water quality, contaminant levels or habitat alterations. Photoautotrophic organisms are found on the surface of built or natural hard substrata such as sand, rocks, stone sculptures, concrete walls and building facades (Gaylarde *et al.*, 2012; Hallmann *et al.*, 2013). For example, in association with other organisms, it was shown that subaerial algae, which can form phototrophic biofilms, contribute to biogenic weathering and to the degradation of the built environment (Crispim *et al.*, 2003; Scheerer *et al.*, 2009; Sterflinger and Pinar, 2013; Pinna, 2014; Ling *et al.*, 2015).

Cities also present a large diversity of built and vacant landscapes (Rupprecht *et al.*, 2015), with a number of informal urban surfaces and area (building gaps, vacant lots, street verges, watersides, walls, fences, roofs …) that have been less investigated in terms of their microbial biodiversity, connectivity, and putative ecological roles. In particular, gutters, which are functionally important structures found nearly everywhere, have been largely overlooked. References to gutters send us back more than two centuries, with birth of the microbiology and the first descriptions of ‘Animalcula’ by Leeuwenhoek in the gutters of houses (Hoole, 1800). Beside the rain gutters found on houses and buildings, these surface water collection channels are also present in the street of essentially all cities in the world. Street gutters have a long and historical existence that spans several centuries. For instance, the Romans started to care about pollution in the streets and modified road-building by digging gutters in the middle of the streets to carry away wastes.

Currently, street gutters, which are most of the time in the sides of the streets, are principally designed for collecting and conveying dirt, litter, debris, storm water and other cast-off waste in lieu of a municipal sanitation system (Selbig, 2016). Most cleaning techniques are manual or mechanized sweeping and can use non-potable water. In fact, some large metropolitan areas and smaller cities have either historical, newly developed, or plan to supply water for the purpose of street sanitation, cleaning or park watering, *etc*. However, depending on the supplied water, street texture, cleaning frequencies and use, and anthropic litter, microbial biofilms/mats develop and might form specific communities with potentially huge public health consequences. The management of street micro-organisms, which might spread within the entire cities, could also have implications in the management of runoff by municipalities. Here, we present at the city scale the first geospatial molecular inventory of the eukaryotic micro-organisms present in the street gutters of Paris. We also compare the street gutters’ eukaryotic photoautotrophic and heterotrophic communities to the ones of the non-potable water sources.

## Materials and methods

### Description of the water supply networks

**Figure 1:**
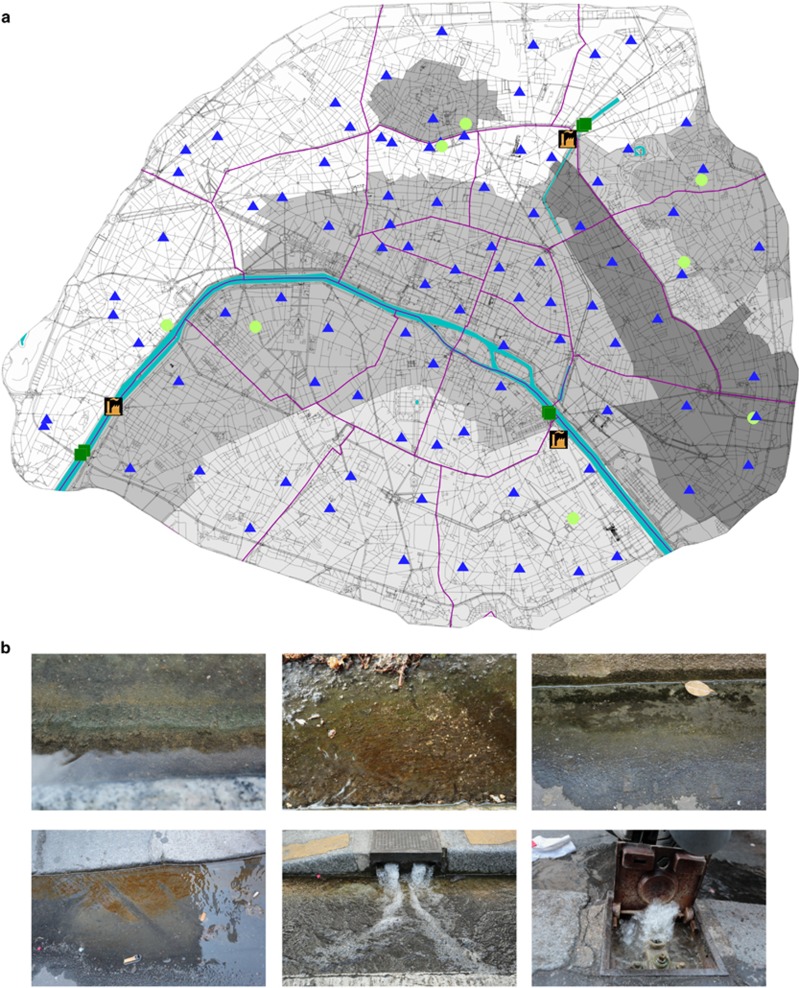
Microbial inventory of the non-potable water networks and street gutters of Paris. (**a**) Geospatial distribution of the sampling sites over the entire Parisian metropolitan area. The color code corresponds to gutter mats (GM, blue, *n=90*), raw water collected from the non-potable subnets (RW, red, *n=8*) and flowing water (FW, orange, *n=6*), which corresponds to the Seine River and a Marne tributary that are used to generate raw water. The grey-scale indicates the seven non-potable water functional subnets (Passy, Montmartre, Ménilmontant, Belleville, Charonne, Villejuif and Bas-Ourcq) in Paris. The orange and black pictograms indicate the location of the three non-potable water factories that are located close to the Seine River (Auteuil and Austerlitz) and to the canal de l’Ourcq (Villette); (**b**) Pictures of the gutters and non-potable water devices sampled across the entire city of Paris. In many cases, microbial mats were clearly visible.

The non-potable water used for the watering of street pavement and gutters originates from three factories. Two are located close to the Seine River (that is, the Austerlitz and Auteuil factories) and the third one close to a river tributary of the Marne, the canal de l’Ourcq (that is, the Villette factory) (Figure 1a). At these factories, the water pumped from these rivers (hereafter named flowing water or FW) goes through stages of screening and grit removal in order to remove fragments and particles through 1 mm^2^. This non-potable water is then stored into seven reservoirs and/or directly used for street sanitation. More specifically, these seven reservoirs will supply seven water subnetworks (Figure 1a). The non-potable water provided by these subnets corresponds to what we named the raw water (RW). Together, FW and RW correspond to the non-potable water sources (NPS).

### Field sampling strategy and procedures

To sample each of the twenty districts of Paris, we first created five street iso-density sectors and geolocalized the barycenter of these sectors using Geoconcept 7.3. The sampling of the street gutters was performed either at these ‘theoretical’ sites or by randomly investigating neighboring streets for the presence of water with or without visible biofilms/mats. These gutter mat (GMs) samples were collected by scrubbing the street surface using a toothbrush and pumping non-potable running or stagnant water. The sampling of the street gutters was performed at the beginning of June 2015 over a 12-day period without any precipitation. Three gutter sites were also sampled during a dry period at the end of July 2015, ending in a total of 90 GM samples.

During the same period in June, we also collected samples in duplicate at a depth of about 1 m in the Seine and in the canal de l’Ourcq, at sites close to the three factories (six samples of FWs). The non-potable water samples from the seven subnets were taken directly out of the watering devices (Figure 1b), that is to say, prior to any contact with gutters or street pavement. In total, the eight RW samples correspond to seven samples from June and one from July 2015. All collected samples were transported to the laboratory within a few hours. Each geotagged site was photo-documented. The environmental samples were homogenized on a rotating wheel, pre-filtered using a 300-μm mesh to remove putative debris, and then filtered through a 5-μm TMTP filter (Millipore, Merck, France). The filters were stored at −80 °C until they were used.

### DNA preparation and sequencing

Total environmental DNA was extracted from the filters of each sample using a PowerBiofilm DNA Isolation kit according to the manufacturer’s procedure (MO BIO, Qiagen, CA, USA). To prepare the 18S rRNA gene libraries, PCR amplifications were performed with primers that contained eight bases at the 5′ end preceded by 2–4 random bases. The V4 region of the 18S rRNA gene region was amplified using the D512for (5′-NtagATTCCAGCTCCAATAGCG-3′) and D978rev (5′-NtagGACTACGATGGTATTAATC-3′) primers, which were initially designed to access in environmental studies the biodiversity of diatoms along with other micro-eukaryotes (Zimmermann *et al.,* 2015). We first performed the PCR amplification using 1 μl of DNA (5–10 ng) in the following mix: 1.00 μl DNA, 1.00 μl Forward Primer (10 μm), 1.00 μl Reverse Primer (10 μm), 0.75 μl DMSO, 0.25 μl BSA (10x), 8.50 μl H_2_O and 12.50 μl PCR Master Mix 2x (KAPA2G Robust HotStart DNA polymerase ReadyMix, KAPA Biosystems, Sigma-Aldrich, France). The following amplification program was used: 95 °C for 5 min; 30 cycles of 95 °C for 15 s, 52 °C for 15 s, and 72 °C for 30 s; and 72 °C for 3 min. The PCR products were checked on an agarose gel, purified using Agencourt AMPure XP beads (Beckman Coulter, High Wycombe, UK), and quantified using a Qubit dsDNA HS assay kit. They were then normalized and pooled (2 pools). We prepared the libraries using 1 μg DNA from the pools and the Illumina TruSeq DNA PCR-Free Library Preparation Kit (Illumina France SARL, San Diego, CA, USA). We followed the supplier's protocol, with the exception that we used a modified End-Repair mix to avoid the production of chimeric constructs. The resulting libraries were quantified by qPCR and sequenced using a MiSeq 2x300 paired-end run, as described by Illumina.

### Sequence processing

Amplicon sequences were analyzed with the *mothur* software version 1.36.1 (Schloss *et al.*, 2009). Reads were processed largely following the Schloss standard operating procedure for MiSeq Illumina data (Kozich *et al.*, 2013). First, contigs between read pairs were assembled, resulting in 10 780 918 raw paired-end DNA sequences. Then, barcode and primer sequences and low-quality sequences were removed (minimum length of 350 bp, maximum length of 460 bp, removing any sequences with ambiguous bases and removing any sequences with homopolymers longer than 8 bp). Subsequently, sequences were aligned to the SILVA reference database release 119 (Quast *et al.*, 2013) and preclustered (pre.cluster, diffs=1). Singletons were excluded (2 399 533 singletons, corresponding to 34.91% of the sequences in this step), and chimeras were removed with the chimera.uchime *mothur* command (217 551 chimeric sequences, corresponding to 4.86% of the sequences in this step). Then, sequences were classified using the k-nearest neighbor (*knn*) algorithm implemented in *mothur* and the BLASTN search method with the SILVA reference database release 119 (Quast *et al.*, 2013). Classification with the *knn* algorithm was chosen because this method was more accurate than the more commonly used naïve Bayesian classification method (Wang *et al.*, 2007) for our data. After classification, non-eukaryotic, unknown and unclassified sequences were excluded (that is, 1 033 123 sequences). To account for differences in sampling efforts, 5638 sequences were then randomly subsampled from each sample. Operational taxonomic units (OTUs) were generated using the average neighbor algorithm. An OTU was defined at the 99% sequence similarity level. After subsampling, the data set corresponded to 586 352 sequences with an average length of 398 bp. These sequences were clustered into 6900 OTUs. Finally, sequences were classified using the *knn* algorithm implemented in *mothur* and the BLASTN search method with the PR2 database (cut-off of 80%) (Guillou *et al.*, 2013). The raw sequence data have been deposited in the NCBI Sequence Read Archive under the BioProject: PRJNA316490.

### Diversity and statistical analysis

Rarefaction curves (calculated from 10 000 iterations; Supplementary Figure 1), diversity indices, Venn diagrams and relative abundances of OTUs were computed with *mothur* (Schloss *et al.*, 2009). All statistical analyses were computed using R software version 3.2.2 (https://www.r-project.org/). After normalization by random subsampling (5638 sequences in each sample, corresponding to the lowest number of sequences in a sample), the OTU matrix was log(1+*x*) transformed, and a Bray-Curtis dissimilarity matrix was computed using the *vegan* package (Oksanen *et al.*, 2015). Global non-metric multidimensional scaling (GNMDS) was computed using the same package based on the Bray-Curtis distances.

The genetic diversity between the different locations was evaluated using analysis of molecular variance (AMOVA) and homogeneity of molecular variance (HOMOVA), which were both based on 1000 iterations and computed with *mothur*. Additionally, eukaryotic 18S rRNA gene sequences aligned in *mothur* were used to compute a neighbor-joining phylogenetic tree with Clearcut (Sheneman *et al.*, 2006). Subsequently, this tree was used to compute phylogenetic distance matrices based on the unweighted and weighted UniFrac algorithms (Lozupone and Knight, 2005) implemented in *mothur* with 1000 iterations.

To investigate the potential presence of compartment-specific OTUs (biomarker) in the studied compartments (non-potable water sources (NPS) and gutter mat (GM)), the linear discriminant analysis (LDA) effect size (LEfSe) algorithm (Segata *et al.*, 2011) implemented in *mothur* was used. Briefly, the algorithm first applies the non-parametric factorial Kruskal–Wallis sum-rank test to detect features with significant differential abundance (*P*<0.05) with respect to the class of interest (here NPS and GM); biological significance is subsequently investigated using a set of pairwise tests among subclasses using the (unpaired) Wilcoxon rank-sum test (*P*<0.05). Finally, the algorithm uses LDA to estimate the effect size of each differentially abundant feature. In the present study, only OTUs presenting an LDA score>log_10_(3) and a significant threshold of *P*<0.05 were considered as biomarkers of a class, that is, a compartment.

To evaluate the co-occurrence probability between all OTUs, the Veech probabilistic model of species co-occurrence (Veech, 2013) was applied using the *cooccur* package (Griffith *et al.*, 2016) in R version 3.2.2. Negative and positive co-occurrences (*P*<0.05) were then analyzed according to the taxonomic classifications of the OTUs.

### Clustering analyses

Our objective was to study how species were distributed among samples, that is, whether species tended to overlap coincidentally in the same samples, thereby identifying clusters of samples with similar species composition. Clustering based on species composition are typically performed with β-diversity metrics on which a hierarchical clustering method is applied (for example, Unweighted Pair-Group Method with Arithmetic Mean) to describe the similarities among samples. Here, we used a novel method recently proposed in ecology and biogeography (Sidor *et al.*, 2013; Vilhena *et al.*, 2013; Vilhena and Antonelli, 2015) based on occurrence networks. This method consists in constructing an occurrence network containing both samples and OTUs as ‘nodes’. OTUs are connected to samples in which they have been found with links proportionally weighted to their abundance. Clusters of highly interconnected samples and OTUs can then be detected with a community algorithm. Community detection algorithms identify clusters of nodes that have high intra-group connectivity and low inter-group connectivity. We applied the map equation algorithm (Rosvall *et al.*, 2009; Rosvall and Bergstrom, 2011) (*Infomap* version Nov. 25, 2015), which was recommended as the most suitable algorithm for such bipartite networks (Vilhena and Antonelli, 2015). We graphically represented the network colored according to clusters with Gephi 0.9.1. to focus on the core of the network. We then graphically filtered out all nodes without at least one link greater than or equal to 50, that is, we kept all samples and OTUs containing at least 50 sequences.

## Results and discussion

### Diversity and structure of the microeukaryotic gutter communities in the French metropolis

To perform the molecular inventory of the French metropolitan city, we collected water and biofilms from 90 street gutters (GMs) that cover all of the twenty districts of Paris (Figure 1a) during a period without any precipitation. Street gutters often present colored biofilms/mats, most often brownish in color (Figure 1b), and could correspond to brown microalgae assemblages assumed to be dominated by diatoms. During the same spring period without rain, we also performed sampling of non-potable waters from the primary water supply sources (these 6 samples are named flowing water samples, or FWs), and from the secondary non-potable water sources that are provided by several subnets (these 8 samples are named raw water samples, or RWs) (for further details see Materials and methods).

**Figure 2:**
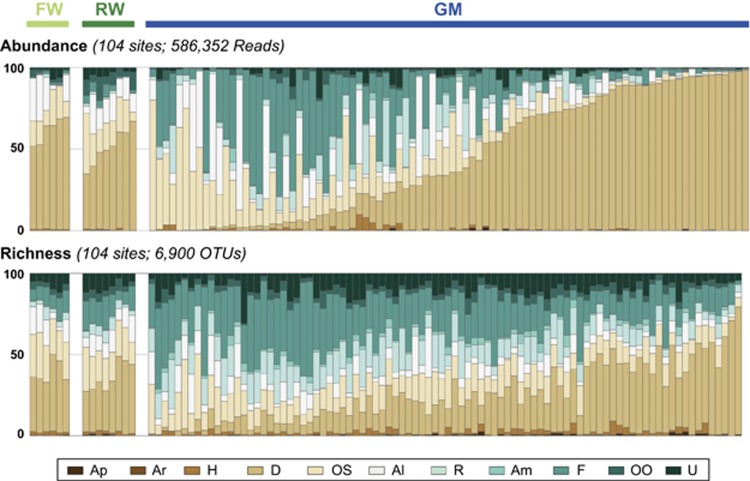
Molecular inventory of the eukaryotic diversity found in Paris. (Upper panel) Relative abundance, and (Lower panel) OTU richness. The code for the different taxonomic assignation is: Amoebozoa (Am), Fungi (F), Other Opisthokonta (OO), Apusozoa (Ap), Archaeplastida (Ar), Hacrobia (H), Diatoms (D), Other Stramenopiles (OS), Alveolata (A), Rhizaria (R), and unclassified (U). The fungi and diatoms are presented independently because they corresponded to significant fractions of Opisthokonta and Stramenopiles, respectively.

Environmental DNA extracted from 104 samples (Supplementary Table 1) was purified and used for PCR amplifications. MiSeq sequencing generated 10 780 918 raw paired-end sequences. After quality filtering and chimera and singleton removal, we finally obtained a total of 6900 operational taxonomic units (OTUs; cut-off 99%) that were taxonomically assigned using the PR2 reference database. The abundance of all OTUs and richness values were calculated for each sampled site (Figure 2).

Because the spring period corresponds to the maximum density of diatoms in the Seine River system (Garnier *et al.*, 1995; Ducharne *et al.*, 2007; Flipo *et al.*, 2007), and part as the consequence of using diatom-specific primers for library construction (see Materials and methods), OTUs assigned to diatoms corresponded to the most abundant fraction in all three compartments (FW, RW and GM), with an average of 61.0% in FW, 50.7% in RW and 43.3% in GM. On average, the second most abundant OTUs group corresponded to the other Stramenopiles (all Stramenopiles lineages but diatoms, named OS), with 15.3% in FW, 18.8% in RW and 15.2% in GM. The third most abundant taxonomic group was the Alveolata, which corresponded to an average of 15.7% in FW, 13.7 in RW, and 11.1% in GM. The other taxonomic groups present a higher variability between compartments, but in the GM the average abundance of the fungi was 18.8, and 6.2% for the Rhizaria. In fact, at most taxonomic levels, the three compartments showed differences in their composition (Supplementary Figure 2).

**Figure 3:**
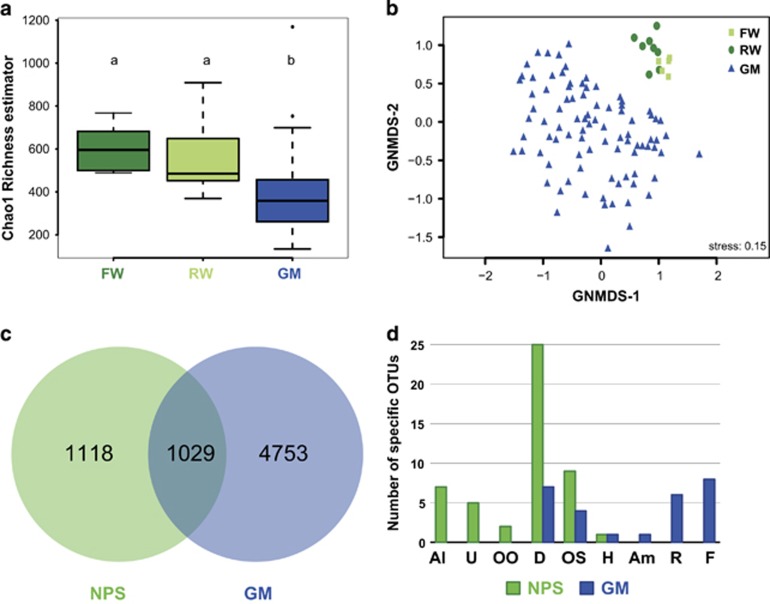
Richness and diversity of the Parisian street gutters and the non-potable sources. (**a**) OTU richness values of the raw water (RW), flowing water (FW) and gutter mats (GM) compartments based on the Chao1 richness estimator. Small letters above the boxplots indicate significantly different distributions (Kruskal–Wallis, *P*<0.05); (**b**) global non-metric multidimensional scaling (GNMDS) ordination of the microbial community composition based on Bray-Curtis distances; (**c**) Venn diagram indicating the specific and/or shared eukaryotic OTUs between the non-potable sources (NPS=FW and RW) and the gutter mats (GM) compartments; (**d**) taxonomic composition of the compartment-specific OTUs identified by LEfSe analysis. Refer to the legend of Figure 2 for the code used for the different taxonomic assignations.

Considering the alpha diversity, a comparison between compartments revealed a significantly lower richness (observed and estimated Chao1 richness) in GMs compared with both FW and RW samples (Kruskal–Wallis, *P*<0.05) (Figure 3a; Supplementary Figure 3a). A more detailed analysis of the alpha diversity using the three Hill’s numbers (Chao *et al.*, 2014) revealed a similar trend between the compartments (Supplementary Figure 3). This indicates that considering either all the analyzed OTUs (Hill number ^0^D, Supplementary Figure 3a) or the dominant OTUs (Hill number ^2^D, Supplementary Figure 3c) results in a similar pattern, suggesting that the reduction of diversity was independent of the OTU size (that is, number of reads within the OTU). Such a reduction of both richness and diversity suggests that the gutters’ niche might act as an environmental filter *stricto sensu* (that is, the abiotic conditions in the GM are the primary explanation of abundance shift (Kraft *et al.*, 2015)) on the aquatic communities from the primary (FW) and secondary (RW) non-potable water sources. Additionally, the niche differences (that is, the nature of the substrate water versus asphalt, concrete and cobblestones), the lifestyle of the community itself (‘free living’ micro-organisms versus adherent cells) and the presence of other residents might also explain the reduction in microbial diversity.

Mineral surfaces usually correspond to a specific habitat with low bioavailable nutrient concentrations and low organic carbon concentrations (Gadd, 2010). Regarding the lifestyle of the communities, the static nature of the biofilms/mats might increase the competition for resources and thus reduce the diversity. Similarly, bacterial richness and diversity have been shown to be significantly lower in stream biofilms compared with the stream water community (Besemer *et al.*, 2012; Wilhelm *et al.*, 2013). Despite this pattern of diversity reduction, a few GM sites (outliers in Supplementary Figure 3) presented a high richness and diversity (for example, site GM_062 showed the highest richness with 729 OTUs, see Supplementary Table 1), indicating heterogeneity in gutter OTU diversity at the city scale. It also suggests the existence of microbial hotspots of diversity in Parisian gutters.

Regarding the beta diversity, we also found significant differences between GM, FW and RW in terms of community composition (Figure 3b), genetic diversity (AMOVA, Fs=25.76, *P*<0.001; HOMOVA, *B*=24.46, *P*<0.001) and phylogenetic diversity (unweighted UniFrac, *P*<0.001; weighted UniFrac, *P*<0.001), indicating that the three studied compartments were distinct. NMDS ordination revealed a clear distinction (that is, no overlap) between the RW and the FW versus the GM communities, indicating that water-associated and gutter-associated microbiomes are dissimilar (Figure 3b). A high similarity among and between the RW and FW samples was also observed (Figure 3b). In particular, FW communities were relatively constrained, with low variation among samples. On the contrary, GM communities showed high variability among samples, highlighting that GM samples can be very dissimilar to each other and that a fraction of the GM samples tended to be more similar to RW or FW samples than to GM samples (Figure 3b). The compositional variability of the GM communities is also reflected by the high taxonomic variability of these communities (Figure 2).

### Compartment-specific microbiomes

As mentioned above, community composition of the primary (FW) and secondary (RW) non-potable water sources show high similarities (Figure 3b). Their small differences are likely to be the result of the griddling process, storage conditions, water circulation, pipes specific organisms, etc. We then compared the street gutter mat (GM) communities to the non-potable water sources (NPS corresponding to FW and RW samples).

Analyses of the OTUs that are specific to the water sources or to the street gutters revealed 1118 OTUs only found in NPS (with 1060 rare OTUs, that is, that have less than 10 sequences), and 1029 shared between NPS and GM (with 381 rare OTUs) (Figure 3c; Supplementary Figure 4). These results indicate that a large proportion of the OTUs present in the incoming waters are able to tolerate the abiotic conditions and the biotic interactions found in gutters. In addition, each one of these two compartments had a high number of exclusive OTUs, with 4753 OTUs (including 3914 rare OTUs) found only in GMs (Figure 3c). In particular, approximately ~54% (645/1196) of the OTUs assigned to diatoms were found to be present only in GMs (Supplementary Figure 4), suggesting that Parisian gutters harbor a rich community of diatoms within their microbial mats. Among the other putative autotrophic algae of the heterokont lineage, we found that ~62% (251/403 OTUs) of Chrysophyceae-Synurophyceae, ~95% (54/57 OTUs) for the Xanthophyceae, and 25% (4/16 OTUs) of the Eustigmatophyceae were only present in GMs but none (0/10 OTUs) of the Dictyochophyceae. Among the large diversity of species present in the GM compartment, we also identified a number of putative microalgae consumers, including Fungi of the phylum Chytridiomycota (Kagami *et al.*, 2012; Rasconi *et al.*, 2012; Frenken *et al.*, 2016), Amoebozoa, and Rhizarian species (specifically members of Cercozoa) (Supplementary Figure 4).

To get a more comprehensive view the organisms that are specific of each of these compartments, we investigated the presence of compartment-specific OTUs using the linear discriminant analysis (LDA) effect size algorithm (LEfSe) that was specifically developed for metagenomic biomarker discovery (Segata *et al.*, 2011). Using LEfSE, 76 compartment-specific OTUs or biomarkers were identified, including 27 OTUs from street gutter and 49 OTUs from the non-potable water source compartments (Figure 3d and Supplementary Table 2). The most represented compartment-specific OTUs corresponded to diatoms. Further phylogenetic analyses revealed that these OTUs are from the three different subclasses of diatoms: Bacillariophycidae, Fragilariophycidae, and Coscinodiscophycidae (Supplementary Table 3). Even if we could not make an identification at the genera level by phylogenetic analyses it is possible that the seven GM-specific OTUs correspond to raphid diatoms that are usually adherent cells and which possess some dispersion capabilities (Svensson *et al.*, 2014). Combined with the 645 OTUs that were only found in GMs, our results suggest that the non-potable sources might contain a pool of potential colonizing diatom species that might be found in the street gutters, where a combination of both abiotic and biotic factors will favor their presence. In addition, it is possible that some of the colonizing diatoms have an aerial or anthropogenic origin, which might not be that surprising given the diversity of substrates colonized by diatoms and other phototrophic organisms (Alfinito *et al.*, 1998; Nowicka-Krawczyk *et al.*, 2014; Piano *et al.*, 2015; Tesson *et al.*, 2016). Finally, we cannot exclude that some of these biofilms/mats might have been initially formed at a different period of the year, even if streets are often swept. To date, based on their morphological characteristics, only 331 diatom taxa had been described throughout the entire Ile-de-France river network (DRIEE, 2013). Thus, our molecular inventory suggests that diatom biodiversity in urban environments is very likely to be rich and needs further investigation, knowing their functional roles and implications in community structuring in freshwaters and biofilms (Battin *et al.*, 2003, 2016; Widder *et al.*, 2014; Velthuis *et al.*, 2017). Interestingly, among the Bacillariophyta-related OTUs that were specific to the NPS compartment, one OTU was found to be homolog (100% identity over 403 nt) to the 18S sequence from a diatom endosymbiont of a freshwater dinoflagellate *Peridiniopsis jiulongensis* (You *et al.*, 2015). Endosymbiotic association within protists (Nowack and Melkonian, 2010) and dinoflagellates (Hehenberger *et al.*, 2016) have been described by their potential to acquire novel biochemical functions. Preliminary analyses of the height Dinophyceae-related OTUs revealed that the most abundant one probably corresponds to an organism of the *Peridiniopsis* genera (not shown).

Interestingly, among the 27 OTUs that were specific to the GM compartment, we also identified eight of them assigned to Fungi. Four OTUs were assigned to the order Chaetothyriales (Ascomycetes) which are often referred to as black yeasts. Chaetothyriales are also known as saprobes on decaying plant matters, but can also be epiphytes, symbiotic or even opportunistic human pathogen (Hubka *et al.*, 2014). Chaetothyriales taxa can be found in various environments, from nutrient-poor substrates (such as rock surfaces and monuments) to humid indoor environments (Isola *et al.*, 2016; Reblova *et al.*, 2016). Three OTUs were related to Dothideomycetes that represent a large and diverse array of fungi in which prominent plant pathogens are over-represented, and also includes numerous rock-inhabiting fungi (Ruibal *et al.*, 2009). Another one of the Fungi-related OTU is likely to correspond to *Cryptococcus* spp., which are widespread fungi in many parts of the world. Among this genus, some species are human pathogens such as *C. neoformans* which is responsible for cryptococcosis (Taylor-Smith and May, 2016).

Another one-fourth (6/27) of the GM-specific OTUs corresponded to Cercozoan (Rhizaria) species. Cercozoans are abundant and therefore ecologically significant soil, freshwater, marine and leaf-associated protists that have roles as bacterivores, as freshwater algae grazers and as parasitoids (Bass and Cavalier-Smith, 2004; Howe *et al.*, 2009; Hess and Melkonian, 2013; Ploch *et al.*, 2016). When considering all of the OTUs present only in the GM compartment, we found that a large number (392/722 OTUs) of the Cercozoan OTUs belonged to the Glissomonadida families, demonstrating the rich diversity of nanoflagellates in street gutters. Three OTUs that were specific to the GMs correspond to sequences related to ‘Spumella-like’ flagellates (Chrysophyceae/Synurophyceae), which are also bactivorous grazers (Grossmann *et al.*, 2016). Finally, one GM-specific OTU corresponded to a putative free-living amoebae that most likely belongs to the family of Hartmannellidae and probably to the species *Hartmannella vermiformis*/*Vermamoeba vermiformis* (best Blast hits). This family of amoebae is widespread in natural or built environments (including water bodies such as hospital water networks) and corresponds to common hosts of human pathogens (Zbikowska *et al.*, 2013; Pagnier *et al.*, 2015).

In the non-potable water sources, we found seven Chrysophyceae/Synurophyceae-related OTUs, and five OTUs related to Suctorian ciliate (Phyllopharyngea, Ciliophora). Suctorians, which are considered to be ecto-commensal organisms, are found in all types of aquatic habitats. They are also considered as epibionts living on different species from small crustaceans to large turtles, plants (Marino-Perez *et al.*, 2010), algae (Moawad, 2010) and Cnidaria (Tazioli and Di Camillo, 2013). One OTU is related to the genus *Dysteria* (Phyllopharyngea, Ciliophora), a group of cyrtophorids that has been found worldwide in the periphyton or as ecto-commensal (Qu *et al.*, 2015). Another OTU, assigned to the clade MAST-12, corresponded to basal heterotrophic Stramenopiles that are distributed in a set of polyphyletic ribogroups collectively named MAST (marine stramenopiles), which have been found in both marine and freshwater environments (Massana *et al.*, 2014; Simon *et al.*, 2015). Another OTU presented a high similarity to the sequence of *Pythium* (Oomycetes, Stramenopiles) genera, known to be putative parasite of plants and animals (Jiang and Tyler, 2012).

Intriguingly, among the non-potable sources specific sequences, we also found invertebrate-related sequences. In particular, one OTU could be assigned to the freshwater sponge genus *Ephydatia* (Spongillina, Demosponges), and probably to *Ephydatia fluviatilis* (best BLAST hits with the GenBank nr/nt database). *Ephydatia*, in particular *E. fluviatilis*, is a relatively common and widely distributed species in rivers and lakes, where it can be found attached to growing mussels, rocks or bridges. The presence of *Ephydatia* in the water used for street sanitation is likely to correspond to the gemmule forms, which are resistant spore-like structures that develop in winter and then later develop into new sponge tissues (Loomis, 2010). One NPS-specific OTU corresponded to a bivalve of the order Veneroida (Heterodonta, Bivalves), and more precisely of the genus *Dreissena* (best BLAST hits with the GenBank nr/nt database) that are mussels frequently found in French rivers (Marescaux *et al.*, 2016). We hypothesize that the sequence from bivalves might have amplified at a larval stage. Altogether, our results demonstrate the presence of a specific diversity of photoautotrophic and heterotrophic micro-eukaryotes and of distinct life stages of multicellular organisms.

### Parisian gutter microbiomes: an everlasting ecological succession?

We investigated the existence of species interactions with co-occurrence analyses and identified numerous correlations between the presence of several taxa. Using all samples and OTUs, we identified 52 543 positive and 18 505 negative significant (*P*<0.05) co-occurrences (Supplementary Tables 4 and 5), with a large proportion corresponding to interactions between Stramenopiles and other taxa, suggesting non-random patterns of assemblages among micro-organisms. In particular, other Stramenopiles (OS) species were likely to occur with Fungi (6.00% of positive co-occurrences) and diatom species (9.26% of positive co-occurrences). We also found positive co-occurrences within diatoms (12.23%), and negative co-occurrences with Fungi (22.15%), other Stramenopiles (11.49%) and Rhizaria (10.08%). Associations between Fungi and diatoms have already been reported in mesotrophic and eutrophic freshwater lakes (Ishida *et al.*, 2015) and marine environments (Scholz *et al.*, 2016). We believe that species interactions could be particularly favored in the biofilms/mats of street gutters. Biotic interactions and pathogenicity within protists and in particular with the diatoms should be further explored.

To further describe aquatic and gutter microbiomes, we grouped samples into homogeneous clusters according to their taxonomic composition, which is a common procedure in ecology and biogeography. We applied a method that was recently proposed in biogeography (Sidor *et al.*, 2013; Vilhena *et al.*, 2013; Vilhena and Antonelli, 2015) and borrowed from network science. The main advantages of this network approach compared with a hierarchical ascendant classification are as follows: (i) species identity is not lost; that is, we can map the connection between samples using species; (ii) clusters are composed of both samples and species; that is, we can describe which taxa are driving clusters; (iii) the results are very similar to clusters based on UPGMA classifications except that ‘transition’ communities between two clusters are not arbitrarily assigned to one of the two clusters but are classified as distinct transition clusters (Vilhena and Antonelli, 2015; Bloomfield *et al.*, 2017); (iv) the network maps the connections between clusters and thus enables a detailed analysis of how our samples are structured; and (v) the map equation algorithm takes into account link weights and thus allows us to include species abundance in the clustering analyses (Rosvall and Bergstrom, 2008). Since the map equation algorithm maximizes intra-group connectivity and minimizes inter-group connectivity, clusters are driven by abundant species, not rare species. We therefore extended the occurrence-based networks (proposed by Vilhena and Antonelli, 2015) to abundance-based networks in this study.

**Figure 4:**
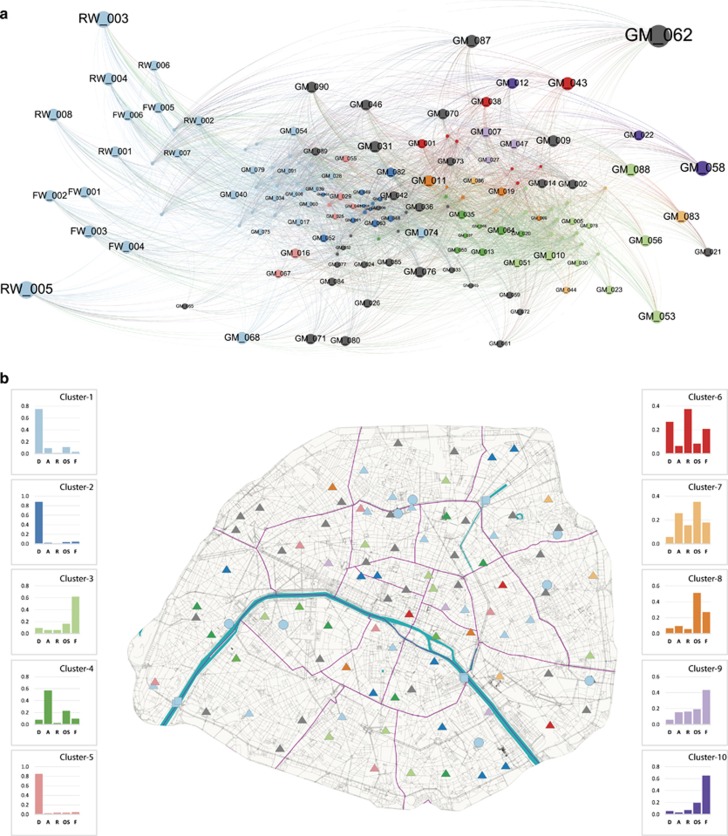
Network analyses of microbiomes in Paris. (**a**) Bipartite network of 104 samples from non-potable sources and street gutter mats of Paris and their most abundant OTUs. Named nodes are samples (GM_001 to GM_90, Gutter Mats; FW_001 to FW_006, Flow Waters; RW_001 to RW_008, Raw Waters), and unnamed nodes are OTUs. The OTUs are connected to the sampling site at which they were identified. The node size is proportional to the weight of links connected to this node. Only the OTUs containing at least 50 sequences are presented. The network was generated using Gephi 0.9.1 with the Force Atlas 2 algorithm. (**b**) Map of Paris showing the different clusters or singletons identified by occurrence network analyses. Bar plots represent the relative abundances of the five main taxonomic groups (covering 74.0% of the total abundance) of the ten largest clusters (no abundance filter). The bar plot abbreviations are as follows: D, Diatoms; A, Alveolata; R, Rhizaria; OS, other Stramenopiles; F, Fungi.

We found 37 clusters in the network of sampling sites and OTUs, with 13 clusters containing more than one gutter sample (Supplementary Table 1). Our most prominent finding was that non-potable sources (flowing and raw waters) were clustered with 14 gutter samples (Figure 4a), indicating that these gutters had similar levels of OTU composition and abundance to their sources (Figure 4b). Then, we observed a clear transition from sources and similar clusters that were dominated by diatoms (clusters 1, 2, 5 and smaller clusters) to compositionally distinct clusters dominated mainly by Alveolata (cluster 4), Fungi (clusters 3, 9 and 10), Rhizaria (cluster 6) or other Stramenopiles (clusters 7 and 8). Interestingly, the transition in mat species composition seemed not to be correlated with geographical location, as sites grouped into a single cluster were distributed throughout the entire city (Figure 4b).

Our analysis of the network of communities provide insights into the structure of microbial communities of street gutters and into the processes underlying their community assemblages. We showed that communities were clustered in groups of similar composition and abundance of OTUs. Interestingly, we found that sources of water (FW and RW) were clustered together with a number of street gutters. This pattern illustrates the important role of water sources in the colonization process of street gutters: source communities are dominated by abundant diatom OTUs, and these diatoms probably colonize gutters when water is used to clean the streets. Then, we identified a transition from gutters similar to the water sources to increasingly distinct gutters, with a diversity of compositions characterized by different dominant taxa (Alveolata, Rhizaria, other Stramenopiles or Fungi). Furthermore, we found that spatial proximity did not explain the ecological similarity of communities, as clusters were scattered throughout the city. The lack of geographical coherence for similar communities could be explained by the existence of microhabitats that drive community similarity across space (Aguilar and Lado, 2012).

A possible alternative hypothesis to explain this lack of geographical coherence in the distribution of clusters may be a temporal explanation; that is, that gutters are subject to an ecological succession from sources to compositionally distinct communities. Gutters are subject to regular disturbances removing communities via mechanic cleaning and flooding from sources. This disturbance has the potential to initiate primary succession where gutters are colonized by species from flooding sources. Then, on a local level, the community diversity might be affected not only by colonization patterns but also to mass effect, local temporal dynamics, and biotic interactions. Communities could also be colonized by a variety of organisms (probably with a diversity of origins), including aero-terrestrial phototrophic and heterotrophic micro-organisms (Polo *et al.*, 2012; Yooseph *et al.*, 2013) driven by human waste (Newall and Walsh, 2005) or waste of animal/pet origin. As communities diversify, other processes might become important, and probably both deterministic and stochastic processes would shape community structures with different trajectories, as demonstrated for other microbial communities (Dumbrell *et al.*, 2010; Shafquat *et al.*, 2014). Future research should concentrate on the monitoring on how gutter communities change in composition through time to deepen our comprehension on the observed patterns of community diversity and composition. A better characterization of the environmental constraints and of the other micro-organisms (for example, bacteria) will allow us to better understand and model network connectivity and species interactions within such freshwater aquatic ecosystem (Verreydt *et al.*, 2012; Besemer *et al.*, 2013; Seymour and Altermatt, 2014).

Overall, our analyses of the network of communities revealed several types of communities with a clear transition from communities of the non-potable water sources to a diversity of compositionally distinct communities in street gutters. Intriguingly, community composition did not appear to be spatially structured, that is, geographically close communities were not necessarily similar. Our data suggests a new pattern in urban biodiversity, with a high level of species only found in street gutters as well as a continuum in the community composition from the sources of non-potable waters to their actual state in streets.

## Conclusions

We reported here for the first time that street gutters harbor an extremely diverse community of eukaryotic microbes with varying patterns of OTU composition, abundance and species exclusive to one compartment. The high levels of co-occurrences between and among heterotrophic (such as fungi) and photoautotrophic (such as diatoms) OTUs found in street gutters suggest the existence of putative trophic dependences in these complex communities, and potential organic matter flows. Interactions at the community level might have potentially important yet disregarded ecological roles in cities.

Important challenges remain associated with understanding the processes that structure gutter communities, such as study of the temporal evolution of street gutter communities, and comparison of these communities between different megapolises/cities across the world. We would like to propose that eukaryotic micro-organisms in street gutter biofilms/mats are likely to be important actors in the treatment of rain water, human waste, pollutants from vehicles and vehicle emissions (tire tread debris, exhaust, motor oil, brake linings, CO_2_, and so on) (Churkina, 2016). These biofilms/mats may also contribute to the decomposition of solid-waste material or street litter (Hobbies *et al.*, 2014), and therefore favor downstream wastewater treatment.

Street gutters harbor and might facilitate, the dispersal of microbes, including parasites and potential pathogens of plants, animals and humans, with possible public health consequences. Understanding street gutter communities’ composition and dynamic is an exciting new topic that might concern most of the urban and peri-urban areas worldwide. In-depth knowledge on the street gutters that could provide ecosystem services, should be used to guide careful planning and targeted action about street cleaning, and sensitize the public and tourists to the presence of a microalgae life in cities.
