# Supplementary material for: Aquatic urban ecology at the scale of a capital: community structure and interactions in street gutters
Source: ISME J. 2017 Oct 13;12(1):253–66. doi: 10.1038/ismej.2017.166 (PMC5739019; doi:10.1038/ismej.2017.166)
Supplement: Supplementary Table 1 [file ismej2017166x1.docx]

**Supplementary Table 1 | Information on the sampling sites.**

| **Names** | **Sample** | **Month** | **Year** | **Address** | **District** | **Subnet** | **Cluster** | **coverage** | **Chao1** | **^0^D (obs)** | **^1^D (Exp. Shannon)** | **^2^D (Inv. Simpson)** | **pH** | **Conductivity** |
| --- | --- | --- | --- | --- | --- | --- | --- | --- | --- | --- | --- | --- | --- | --- |
| FW_001 | FW | June | 2015 | Quai Saint-Bernard | 75005 | NA | 1 | 0,971 | 590,3 | 335 | 49,2 | 23,6 | 8,33 | 528 |
| FW_002 | FW | June | 2015 | Quai Saint-Bernard | 75005 | NA | 1 | 0,970 | 601,1 | 355 | 51,3 | 24,0 | 8,32 | 827 |
| FW_003 | FW | June | 2015 | Voie Georges Pompidou | 75016 | NA | 1 | 0,967 | 681,8 | 380 | 44,4 | 14,6 | 8,20 | 553 |
| FW_004 | FW | June | 2015 | Voie Georges Pompidou | 75016 | NA | 1 | 0,966 | 767,8 | 373 | 37,4 | 11,0 | 8,26 | 537 |
| FW_005 | FW | June | 2015 | Quai de Loire | 75019 | NA | 1 | 0,973 | 499,7 | 317 | 23,1 | 6,8 | 8,37 | 697 |
| FW_006 | FW | June | 2015 | Quai de Loire | 75019 | NA | 1 | 0,975 | 489,4 | 304 | 23,2 | 7,0 | NA | NA |
| GM_001 | GM | June | 2015 | 2-4 Rue du Plat d'Étain | 75001 | Bas-Ourcq | 6 | 0,977 | 553,7 | 269 | 36,6 | 14,0 | 8,10 | 742 |
| GM_002 | GM | June | 2015 | 3 rue de Montesquieu | 75001 | Bas-Ourcq | 26 | 0,982 | 360,2 | 278 | 28,6 | 9,4 | 7,97 | 717 |
| GM_003 | GM | June | 2015 | 22 Place Vendôme | 75001 | Bas-Ourcq | 2 | 0,993 | 147,7 | 93 | 6,8 | 4,0 | 8,16 | 685 |
| GM_004 | GM | June | 2015 | 15 rue Dalayrac | 75002 | Bas-Ourcq | 2 | 0,992 | 134,1 | 81 | 5,3 | 3,5 | 8,15 | 710 |
| GM_005 | GM | June | 2015 | 9 rue Tracy | 75002 | Bas-Ourcq | 3 | 0,988 | 256,6 | 204 | 23,6 | 11,3 | 8,18 | 682 |
| GM_006 | GM | June | 2015 | 31 rue Montmartre | 75002 | Bas-Ourcq | 18 | 0,991 | 238,9 | 123 | 8,4 | 4,0 | 8,18 | 471 |
| GM_007 | GM | June | 2015 | 53 rue des Archives | 75003 | Bas-Ourcq | 9 | 0,979 | 428,7 | 303 | 49,8 | 18,9 | 8,22 | 725 |
| GM_008 | GM | June | 2015 | 74 rue Turenne | 75003 | Villejuif | 1 | 0,989 | 231,6 | 151 | 4,1 | 1,8 | 7,94 | 560 |
| GM_009 | GM | June | 2015 | 8 Cité Dupetit-Thouars | 75003 | Bas-Ourcq | 25 | 0,978 | 475,0 | 348 | 59,2 | 22,8 | 7,93 | 787 |
| GM_010 | GM | June | 2015 | 3 Rue Cunin-Gridaine | 75003 | Bas-Ourcq | 3 | 0,985 | 418,0 | 308 | 65,5 | 27,6 | 8,00 | 793 |
| GM_011 | GM | June | 2015 | 16 place des Vosges | 75004 | Bas-Ourcq | 8 | 0,975 | 517,1 | 353 | 56,7 | 19,9 | 8,31 | 703 |
| GM_012 | GM | June | 2015 | 90 rue de l'Hôtel de ville | 75004 | Bas-Ourcq | 10 | 0,977 | 486,7 | 320 | 43,5 | 12,6 | 8,23 | 695 |
| GM_013 | GM | June | 2015 | 271 rue Saint Jacques | 75005 | Villejuif | 4 | 0,984 | 306,1 | 215 | 17,9 | 6,7 | 8,26 | 768 |
| GM_014 | GM | June | 2015 | 24 rue Lhomond | 75005 | Villejuif | 36 | 0,986 | 344,1 | 267 | 41,9 | 16,9 | 8,32 | 705 |
| GM_015 | GM | June | 2015 | 10 Rue des Chantiers | 75005 | Bas-Ourcq | 2 | 0,992 | 155,2 | 79 | 4,5 | 2,8 | 9,07 | 496 |
| GM_016 | GM | June | 2015 | 14 rue des beaux arts | 75006 | Bas-Ourcq | 5 | 0,982 | 373,6 | 285 | 29,4 | 11,3 | 8,24 | 725 |
| GM_017 | GM | June | 2015 | 14 rue de l'Eperon | 75006 | Bas-Ourcq | 1 | 0,983 | 363,5 | 204 | 13,3 | 5,0 | 8,07 | 762 |
| GM_018 | GM | June | 2015 | 5 Rue Joseph Bara | 75006 | Bas-Ourcq | 2 | 0,991 | 207,0 | 109 | 4,5 | 2,1 | 8,28 | 755 |
| GM_019 | GM | June | 2015 | 12 rue de l'Abbé Grégoire | 75006 | Bas-Ourcq | 8 | 0,979 | 501,3 | 251 | 27,9 | 11,1 | 8,24 | 782 |
| GM_020 | GM | June | 2015 | 103 rue de Grenelles | 75007 | Bas-Ourcq | 4 | 0,990 | 230,0 | 173 | 10,7 | 3,5 | 7,99 | 645 |
| GM_021 | GM | June | 2015 | 25-27 rue Oudinot | 75007 | Bas-Ourcq | 14 | 0,983 | 359,6 | 254 | 18,9 | 5,9 | 7,54 | 185 |
| GM_022 | GM | June | 2015 | 11 rue Surcouf | 75007 | Bas-Ourcq | 10 | 0,977 | 467,9 | 295 | 30,5 | 11,8 | 8,35 | 774 |
| GM_023 | GM | June | 2015 | 23 Avenue de la Bourdonnais | 75007 | Bas-Ourcq | 3 | 0,985 | 399,0 | 251 | 40,0 | 17,1 | 7,43 | 1280 |
| GM_024 | GM | June | 2015 | 72 rue du Rocher | 75008 | Passy | 30 | 0,986 | 258,1 | 181 | 15,3 | 7,7 | 8,02 | 633 |
| GM_025 | GM | June | 2015 | 26 rue de Coucelles | 75008 | Bas-Ourcq | 5 | 0,988 | 237,6 | 150 | 8,4 | 3,3 | 7,92 | 471 |
| GM_026 | GM | June | 2015 | 29 rue Washington | 75008 | Passy | 22 | 0,982 | 446,0 | 244 | 29,9 | 11,0 | 8,72 | 750 |
| GM_027 | GM | June | 2015 | 3 rue Montalivet | 75008 | Bas-Ourcq | 9 | 0,987 | 283,1 | 196 | 22,7 | 9,9 | 8,17 | 537 |
| GM_028 | GM | June | 2015 | 15 rue Mansart | 75009 | Passy | 1 | 0,988 | 261,4 | 180 | 9,9 | 3,3 | 8,01 | 695 |
| GM_029 | GM | June | 2015 | 24 rue de Ventimille | 75009 | Passy | 5 | 0,984 | 328,2 | 199 | 13,1 | 5,9 | 8,06 | 686 |
| GM_030 | GM | June | 2015 | 89 rue de la victoire | 75009 | Bas-Ourcq | 3 | 0,991 | 240,4 | 183 | 29,3 | 12,4 | 7,78 | 2644 |
| GM_031 | GM | June | 2015 | 6 rue de Provence | 75009 | Bas-Ourcq | 21 | 0,978 | 469,3 | 352 | 53,7 | 16,4 | 7,95 | 685 |
| GM_032 | GM | June | 2015 | 1 rue Auber | 75009 | Bas-Ourcq | 11 | 0,990 | 202,7 | 120 | 5,1 | 2,3 | 8,01 | 706 |
| GM_033 | GM | June | 2015 | 16 rue Alexandra Parodi | 75010 | Passy | 29 | 0,989 | 231,2 | 158 | 15,0 | 6,0 | 8,10 | 724 |
| GM_034 | GM | June | 2015 | 1 Rue des Messageries | 75010 | Bas-Ourcq | 1 | 0,986 | 265,8 | 170 | 4,9 | 2,2 | 7,89 | 667 |
| GM_035 | GM | June | 2015 | 17 rue demarquay | 75010 | Passy | 4 | 0,981 | 423,2 | 246 | 26,6 | 11,9 | 7,64 | 763 |
| GM_036 | GM | June | 2015 | 24 Rue Lucien Sampaix | 75010 | Bas-Ourcq | 20 | 0,984 | 358,0 | 267 | 39,2 | 14,6 | 8,00 | 672 |
| GM_037 | GM | June | 2015 | 2 rue Tesson | 75010 | Charonne | 4 | 0,990 | 209,6 | 119 | 12,3 | 6,1 | 7,74 | 809 |
| GM_038 | GM | June | 2015 | 102 rue Saint-Maur | 75011 | Charonne | 6 | 0,983 | 375,6 | 297 | 51,1 | 23,3 | 7,97 | 540 |
| GM_039 | GM | June | 2015 | 7 rue Gerbier | 75011 | Charonne | 1 | 0,984 | 312,3 | 164 | 7,4 | 3,8 | 7,68 | 827 |
| GM_040 | GM | June | 2015 | 7 Passage Charles Dallery | 75011 | Bas-Ourcq | 1 | 0,980 | 422,0 | 261 | 15,0 | 4,4 | 8,07 | 685 |
| GM_041 | GM | June | 2015 | 11 rue Moufle | 75011 | Bas-Ourcq | 5 | 0,989 | 222,4 | 141 | 6,9 | 3,4 | 7,95 | 665 |
| GM_042 | GM | June | 2015 | 21 rue de la Véga | 75012 | Charonne | 27 | 0,977 | 456,6 | 269 | 22,5 | 8,9 | 8,08 | 589 |
| GM_043 | GM | June | 2015 | 275 rue de Charenton | 75012 | Charonne | 6 | 0,971 | 577,7 | 402 | 63,4 | 27,6 | 8,10 | 578 |
| GM_044 | GM | June | 2015 | 4 rue Jean Bouton | 75012 | Bas-Ourcq | 7 | 0,984 | 319,2 | 190 | 8,7 | 4,4 | 7,79 | 774 |
| GM_045 | GM | June | 2015 | 10 rue Dorian | 75012 | Charonne | 16 | 0,990 | 177,5 | 126 | 6,2 | 3,4 | 8,13 | 501 |
| GM_046 | GM | June | 2015 | 2 rue de la Voûte | 75012 | Charonne | 33 | 0,971 | 544,5 | 320 | 27,0 | 9,8 | 7,96 | 541 |
| GM_047 | GM | June | 2015 | 4 rue de Bellièvre | 75013 | Villejuif | 9 | 0,981 | 415,8 | 274 | 41,5 | 15,9 | 7,95 | 324 |
| GM_048 | GM | June | 2015 | 17 rue du chevaleret | 75013 | Villejuif | 2 | 0,987 | 277,5 | 168 | 8,7 | 3,3 | 8,00 | 545 |
| GM_049 | GM | June | 2015 | 2 passage Nationale | 75013 | Villejuif | 2 | 0,987 | 265,5 | 146 | 6,5 | 3,5 | 8,04 | 476 |
| GM_050 | GM | June | 2015 | 22 rue Auguste Perret | 75013 | Villejuif | 4 | 0,986 | 294,6 | 170 | 15,3 | 6,6 | 7,77 | 751 |
| GM_051 | GM | June | 2015 | 2 rue Auguste Lançon | 75013 | Villejuif | 3 | 0,986 | 324,5 | 261 | 34,5 | 14,2 | 8,42 | 355 |
| GM_052 | GM | June | 2015 | 14 rue Watteau | 75013 | Villejuif | 2 | 0,983 | 360,8 | 212 | 15,8 | 6,2 | 8,08 | 532 |
| GM_053 | GM | June | 2015 | 17-19 rue de Plaisance | 75014 | Villejuif | 3 | 0,973 | 520,6 | 377 | 49,4 | 17,4 | 8,00 | 572 |
| GM_054 | GM | June | 2015 | 5 Rue Auguste Mie | 75014 | Villejuif | 1 | 0,976 | 472,5 | 270 | 9,2 | 2,9 | 8,17 | 546 |
| GM_055 | GM | June | 2015 | 42 rue des Artistes | 75014 | Villejuif | 5 | 0,983 | 370,3 | 204 | 11,9 | 4,7 | 8,13 | 527 |
| GM_056 | GM | June | 2015 | rue Messier | 75014 | Villejuif | 3 | 0,984 | 412,2 | 333 | 55,8 | 17,4 | 8,14 | 624 |
| GM_058 | GM | June | 2015 | 37 rue Saint Charles | 75015 | Bas-Ourcq | 10 | 0,967 | 699,2 | 466 | 86,1 | 35,1 | 8,05 | 550 |
| GM_059 | GM | June | 2015 | 90 rue Balard | 75015 | Bas-Ourcq | 12 | 0,985 | 303,9 | 187 | 14,4 | 7,2 | 7,75 | 740 |
| GM_060 | GM | June | 2015 | 20 rue Eugène Millon | 75015 | Villejuif | 1 | 0,987 | 276,0 | 162 | 7,5 | 3,8 | 8,06 | 524 |
| GM_061 | GM | June | 2015 | 5-7-9 Place Falguière | 75015 | Villejuif | 28 | 0,982 | 338,8 | 204 | 14,7 | 5,1 | 7,74 | 1189 |
| GM_062 | GM | June | 2015 | 133 Rue Castagnary | 75015 | Villejuif | 31 | 0,943 | 1169,0 | 729 | 183,7 | 69,3 | 8,10 | 543 |
| GM_063 | GM | June | 2015 | 1 rue Mesnil | 75016 | Passy | 2 | 0,986 | 286,0 | 196 | 12,5 | 5,1 | 7,77 | 623 |
| GM_064 | GM | June | 2015 | 4 rue Jean Richepin | 75016 | Passy | 4 | 0,979 | 435,5 | 260 | 21,7 | 7,5 | 7,94 | 567 |
| GM_065 | GM | June | 2015 | 7 rue de la Pompe | 75016 | Passy | 15 | 0,988 | 255,5 | 155 | 13,3 | 6,8 | 8,07 | 549 |
| GM_066 | GM | June | 2015 | 62 Rue Raynouard | 75016 | Passy | 4 | 0,994 | 150,0 | 115 | 6,5 | 2,7 | 7,67 | 619 |
| GM_067 | GM | June | 2015 | 4 rue Michel Ange | 75016 | Passy | 5 | 0,982 | 337,5 | 232 | 13,3 | 4,9 | 7,87 | 552 |
| GM_068 | GM | June | 2015 | 21 rue Michel Ange | 75016 | Passy | 1 | 0,969 | 617,5 | 361 | 26,8 | 8,2 | 7,83 | 575 |
| GM_069 | GM | June | 2015 | 93 rue Nollet | 75017 | Passy | 8 | 0,991 | 191,4 | 132 | 11,0 | 5,0 | 7,80 | 732 |
| GM_070 | GM | June | 2015 | 1 rue Truffaut | 75017 | Passy | 32 | 0,979 | 442,0 | 310 | 53,5 | 25,6 | 8,25 | 666 |
| GM_071 | GM | June | 2015 | 32 boulevard Pereire | 75017 | Passy | 13 | 0,978 | 433,0 | 312 | 33,0 | 13,3 | 7,87 | 567 |
| GM_072 | GM | June | 2015 | 81 avenue Niel | 75017 | Passy | 12 | 0,987 | 252,5 | 170 | 8,8 | 3,4 | 7,85 | 815 |
| GM_073 | GM | June | 2015 | 32 Place Saint-Ferdinand | 75017 | Passy | 17 | 0,987 | 327,0 | 252 | 32,6 | 10,0 | 7,80 | 724 |
| GM_074 | GM | June | 2015 | 29 rue Berthe | 75018 | Montmartre | 1 | 0,974 | 563,8 | 335 | 25,9 | 6,0 | 8,15 | 714 |
| GM_075 | GM | June | 2015 | 27 rue de Maistre | 75018 | Passy | 1 | 0,986 | 329,1 | 171 | 8,1 | 2,8 | 7,87 | 745 |
| GM_076 | GM | June | 2015 | 73 rue Belliard | 75018 | Passy | 35 | 0,977 | 492,9 | 320 | 31,1 | 11,1 | 8,20 | 705 |
| GM_077 | GM | June | 2015 | 9 rue Raymond Queneau | 75018 | Passy | 11 | 0,990 | 219,6 | 156 | 10,2 | 4,4 | 7,95 | 727 |
| GM_078 | GM | June | 2015 | 3 rue Jean Robert | 75018 | Passy | 3 | 0,989 | 226,3 | 154 | 13,1 | 5,3 | 7,85 | 745 |
| GM_079 | GM | June | 2015 | 21 rue Burnouf | 75019 | Menilmontant | 1 | 0,979 | 427,4 | 219 | 6,3 | 2,3 | 7,95 | 698 |
| GM_080 | GM | June | 2015 | 15 rue des Lilas | 75019 | Belleville | 23 | 0,976 | 492,6 | 293 | 21,9 | 6,3 | 8,04 | 683 |
| GM_081 | GM | June | 2015 | 1b rue Curial | 75019 | Passy | 2 | 0,990 | 274,8 | 137 | 11,9 | 6,1 | 8,03 | 710 |
| GM_082 | GM | June | 2015 | 26 rue Cambrai | 75019 | Passy | 2 | 0,975 | 521,3 | 278 | 18,4 | 6,6 | 8,00 | 717 |
| GM_083 | GM | June | 2015 | 151 rue Manin | 75019 | Menilmontant | 7 | 0,974 | 550,4 | 366 | 52,5 | 18,6 | 7,36 | 607 |
| GM_084 | GM | June | 2015 | 59 rue Manin | 75019 | Menilmontant | 13 | 0,981 | 387,9 | 233 | 16,9 | 5,8 | 7,87 | 735 |
| GM_085 | GM | June | 2015 | 93 rue des Cascades | 75020 | Belleville | 19 | 0,984 | 351,6 | 224 | 22,2 | 7,2 | 7,79 | 835 |
| GM_086 | GM | June | 2015 | 18 Rue Alphonse Penaud | 75020 | Belleville | 7 | 0,987 | 266,0 | 191 | 17,4 | 6,9 | 7,79 | 731 |
| GM_087 | GM | June | 2015 | 3 rue des Mûriers | 75020 | Menilmontant | 24 | 0,965 | 752,8 | 391 | 47,4 | 18,3 | 7,93 | 733 |
| GM_088 | GM | June | 2015 | 39 rue de la Plaine | 75020 | Charonne | 3 | 0,970 | 646,6 | 387 | 64,4 | 21,1 | 7,76 | 643 |
| GM_089 | GM | July | 2015 | 1 rue Alfred Stevens | 75009 | Passy | 37 | 0,982 | 395,5 | 226 | 22,6 | 11,3 | 8,00 | 582 |
| GM_090 | GM | July | 2015 | 9 Rue Bochart de Saron | 75009 | Passy | 34 | 0,972 | 587,9 | 338 | 25,9 | 8,0 | 7,86 | 571 |
| GM_091 | GM | July | 2015 | 19, bld Rochechouart | 75009 | Passy | 1 | 0,983 | 425,3 | 172 | 9,8 | 4,1 | 7,86 | 573 |
| RW_001 | RW | June | 2015 | 34 Avenue Bosquet | 75007 | Bas-Ourcq | 1 | 0,982 | 433,0 | 336 | 48,6 | 16,8 | 8,29 | 699 |
| RW_002 | RW | June | 2015 | 1 rue Jean-Baptiste Say | 75009 | Passy | 1 | 0,976 | 496,6 | 291 | 19,4 | 5,8 | 8,29 | 696 |
| RW_003 | RW | June | 2015 | 2 rue de la Voûte | 75012 | Charonne | 1 | 0,962 | 721,2 | 514 | 66,2 | 18,4 | 8,32 | 673 |
| RW_004 | RW | June | 2015 | 32 rue Dunois | 75013 | Villejuif | 1 | 0,979 | 474,4 | 383 | 36,0 | 9,7 | 8,32 | 612 |
| RW_005 | RW | June | 2015 | 3 Rue de l'Alboni | 75016 | Passy | 1 | 0,957 | 909,0 | 546 | 80,3 | 24,8 | 8,32 | 572 |
| RW_006 | RW | June | 2015 | 13 Rue Henri Ribière | 75019 | Belleville | 1 | 0,984 | 369,8 | 309 | 44,0 | 15,3 | 8,31 | 701 |
| RW_007 | RW | June | 2015 | 7 rue Soleillet | 75020 | Menilmontant | 1 | 0,977 | 472,3 | 255 | 19,0 | 7,6 | 8,38 | 696 |
| RW_008 | RW | July | 2015 | 9 rue de Clignancourt | 75018 | Montmartre | 1 | 0,974 | 577,0 | 380 | 75,1 | 30,8 | 8,11 | 555 |
