# Supplementary material for: Aquatic urban ecology at the scale of a capital: community structure and interactions in street gutters
Source: ISME J. 2017 Oct 13;12(1):253–66. doi: 10.1038/ismej.2017.166 (PMC5739019; doi:10.1038/ismej.2017.166)
Supplement: Supplementary Table 3 [file ismej2017166x3.docx]

**Supplementary Table 3 | Phylogeny-based classification of the compartment specific OTUs belonging to *Bacillariophyta.***

| **Class** | **OTU name** | | **Subclass** | | **Order** | | **Family** | **Genus** |
| --- | --- | --- | --- | --- | --- | --- | --- | --- |
| Non-Potable Sources (NPS) | Otu00137 | Bacillariophyceae | | Cocconeidales | | Cocconeidaceae | | Cocconeis |
|  | Otu00147 | Bacillariophyceae | | Cocconeidales | | Cocconeidaceae | | Cocconeis |
|  | Otu00228 | Bacillariophyceae | | Cocconeidales | | Cocconeidaceae | | Cocconeis |
|  | Otu00258 | Bacillariophyceae | | Cocconeidales | | Cocconeidaceae | | Cocconeis |
|  | Otu00149 | Bacillariophyceae | | Cymbellales | | Gomphonemataceae | | Gomphonema |
|  | Otu00182 | Bacillariophyceae | | Cymbellales | | Rhoicospheniaceae | | Rhoicosphenia |
|  | Otu00081 | Bacillariophyceae | | Naviculales | | Naviculaceae | | Navicula |
|  | Otu00013 | Bacillariophyceae | | Surirellales | | Surirellaceae | |  |
|  | Otu00014 | Bacillariophyceae | | Surirellales | | Surirellaceae | |  |
|  | Otu00024 | Bacillariophyceae | | Surirellales | | Surirellaceae | |  |
|  | Otu00152 | Bacillariophyceae | | Surirellales | | Surirellaceae | |  |
|  | Otu00001 | Coscinodiscophyceae | | Melosirales | | Melosiraceae | | Melosira |
|  | Otu00010 | Coscinodiscophyceae | | Melosirales | | Melosiraceae | | Melosira |
|  | Otu00085 | Coscinodiscophyceae | | Thalassiosirales | | Stephanodiscaceae | |  |
|  | Otu00160 | Coscinodiscophyceae | | Thalassiosirales | | Stephanodiscaceae | | Cyclotella |
|  | Otu00196 | Coscinodiscophyceae | | Thalassiosirales | | Stephanodiscaceae | |  |
|  | Otu00268 | Coscinodiscophyceae | | Thalassiosirales | | Stephanodiscaceae | | Cyclotella |
|  | Otu00153 | Coscinodiscophyceae | | Thalassiosirales | | Thalassiosiraceae | | Thalassiosira |
|  | Otu00252 | Coscinodiscophyceae | | Thalassiosirales | | Thalassiosiraceae | | Thalassiosira |
|  | Otu00297 | Coscinodiscophyceae | | Thalassiosirales | | Thalassiosiraceae | | Thalassiosira |
|  | Otu00066 | Fragilariophyceae | | Fragilariales | | Fragilariaceae | | Synedra |
|  | Otu00067 | Fragilariophyceae | | Fragilariales | | Fragilariaceae | |  |
|  | Otu00172 | Fragilariophyceae | | Fragilariales | | Fragilariaceae | | Synedra |
|  | Otu00211 | Fragilariophyceae | | Fragilariales | | Fragilariaceae | |  |
|  | Otu00020 | unclassified-Bacillariophyta | | | |  | |  |
| Gutter Mats (GM) | Otu00002 | Bacillariophyceae | | Bacillariales | | Bacillariaceae | | Nitzschia |
|  | Otu00004 | Bacillariophyceae | | Bacillariales | | Bacillariaceae | | Nitzschia |
|  | Otu00041 | Bacillariophyceae | | Bacillariales | | Bacillariaceae | | Nitzschia |
|  | Otu00017 | Bacillariophyceae | | Naviculales | | Naviculaceae | | Navicula |
|  | Otu00035 | Bacillariophyceae | | Naviculales | | Naviculaceae | | Navicula |
|  | Otu00003 | Bacillariophyceae | | Surirellales | | Surirellaceae | |  |
|  | Otu00039 | Bacillariophyceae | | Thalassiophysales | | Catenulaceae | | Amphora |
