# Supplementary material for: Aquatic urban ecology at the scale of a capital: community structure and interactions in street gutters
Source: ISME J. 2017 Oct 13;12(1):253–66. doi: 10.1038/ismej.2017.166 (PMC5739019; doi:10.1038/ismej.2017.166)
Supplement: Supplementary Table 4 [file ismej2017166x4.docx]

**Supplementary Table 4 | Positive co-occurrences** (*p*-value < 0.05) **between species of the Paris non-drinkable water network.** The values the correspond to percentage from 52,543 co-occurrences, found within the 6,900 OTUs of the 104 environmental samples.

|  | **Amoebozoa** | **Fungi** | **Other-**  **Opisthokonta** | **Apusozoa** | **Hacrobia** | **Diatoms** | **Other-**  **Stramenopiles** | **Alveolata** | **Rhizaria** | **unclassified** |
| --- | --- | --- | --- | --- | --- | --- | --- | --- | --- | --- |
| **Amoebozoa** | 0,06 | 1,03 | 0,11 | 0,02 | 0,08 | 0,15 | 0,41 | 0,10 | 0,68 | 0,34 |
| **Fungi** |  | **9,26** | 1,07 | 0,04 | 0,77 | 3,37 | **6,00** | 1,58 | **9,27** | 4,53 |
| **Other-**  **Opisthokonta** |  |  | 0,14 | 0,04 | 0,17 | 1,69 | 1,28 | 0,47 | 0,61 | 0,50 |
| **Apusozoa** |  |  |  | 0,01 | 0,04 | 0,20 | 0,11 | 0,05 | 0,07 | 0,08 |
| **Hacrobia** |  |  |  |  | 0,08 | 1,04 | 0,76 | 0,27 | 0,53 | 0,39 |
| **Diatoms** |  |  |  |  |  | **12,23** | **9,26** | 3,70 | 2,03 | 3,81 |
| **Other-**  **Stramenopiles** |  |  |  |  |  |  | 3,52 | 2,42 | 3,53 | 2,94 |
| **Alveolata** |  |  |  |  |  |  |  | 0,53 | 0,96 | 0,74 |
| **Rhizaria** |  |  |  |  |  |  |  |  | 3,20 | 2,79 |
| **unclassified** |  |  |  |  |  |  |  |  |  | 0,93 |
