# Supplementary material for: Aquatic urban ecology at the scale of a capital: community structure and interactions in street gutters
Source: ISME J. 2017 Oct 13;12(1):253–66. doi: 10.1038/ismej.2017.166 (PMC5739019; doi:10.1038/ismej.2017.166)
Supplement: Supplementary Table 5 [file ismej2017166x5.docx]

**Supplementary Table 5 | Negative co-occurrences** (*p*-value < 0.05) **between species of the Paris non-drinkable water network.** The values correspond to the percentage from 18,505 co-occurrences, found within the 6,900 OTUs of the 104 environmental samples.

|  | **Amoebozoa** | **Fungi** | **Other-Opisthokonta** | **Apusozoa** | **Hacrobia** | **Diatoms** | **Other-**  **Stramenopiles** | **Alveolata** | **Rhizaria** | **unclassified** |
| --- | --- | --- | --- | --- | --- | --- | --- | --- | --- | --- |
| **Amoebozoa** | 0,00 | 0,32 | 0,06 | 0,00 | 0,09 | 1,35 | 0,49 | 0,21 | 0,21 | 0,15 |
| **Fungi** |  | 1,63 | 1,33 | 0,35 | 1,15 | **22,15** | **7,26** | 3,03 | 2,43 | 2,45 |
| **Other-**  **Opisthokonta** |  |  | 0,07 | 0,01 | 0,22 | 1,49 | 1,09 | 0,34 | 0,49 | 0,28 |
| **Apusozoa** |  |  |  | 0,00 | 0,02 | 0,09 | 0,22 | 0,06 | 0,10 | 0,05 |
| **Hacrobia** |  |  |  |  | 0,06 | 1,30 | 0,87 | 0,36 | 0,64 | 0,22 |
| **Diatoms** |  |  |  |  |  | 1,91 | **11,49** | 4,92 | **10,08** | 3,42 |
| **Other-**  **Stramenopiles** |  |  |  |  |  |  | 3,32 | 2,05 | 3,84 | 1,77 |
| **Alveolata** |  |  |  |  |  |  |  | 0,22 | 1,73 | 0,61 |
| **Rhizaria** |  |  |  |  |  |  |  |  | 0,81 | 1,08 |
| **unclassified** |  |  |  |  |  |  |  |  |  | 0,19 |
