# Supplementary material for: Aquatic urban ecology at the scale of a capital: community structure and interactions in street gutters
Source: ISME J. 2017 Oct 13;12(1):253–66. doi: 10.1038/ismej.2017.166 (PMC5739019; doi:10.1038/ismej.2017.166)
Supplement: Supplementary Figure 1 [file ismej2017166x6.pdf]

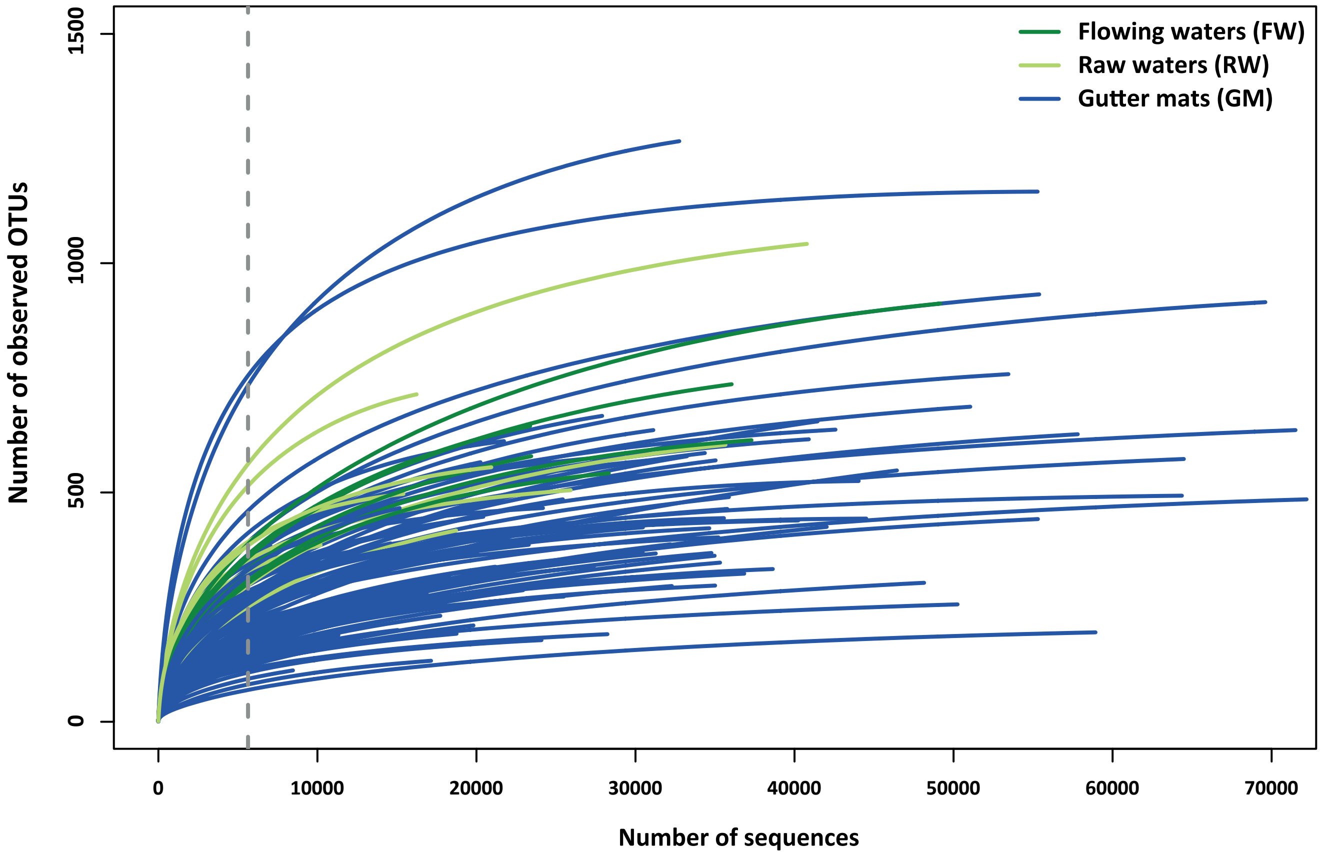

Supplementary Figure 1 | Rarefaction curves for the numbers of observed OTUs defined by at least 99% similarity between 18S rRNA gene sequences for the 104 analyzed samples. To account for differences in sampling efforts, 5,638 sequences (vertical dotted line) were randomly subsampled from each sample.
