# Supplementary material for: Aquatic urban ecology at the scale of a capital: community structure and interactions in street gutters
Source: ISME J. 2017 Oct 13;12(1):253–66. doi: 10.1038/ismej.2017.166 (PMC5739019; doi:10.1038/ismej.2017.166)
Supplement: Supplementary Figure 2 [file ismej2017166x7.pdf]

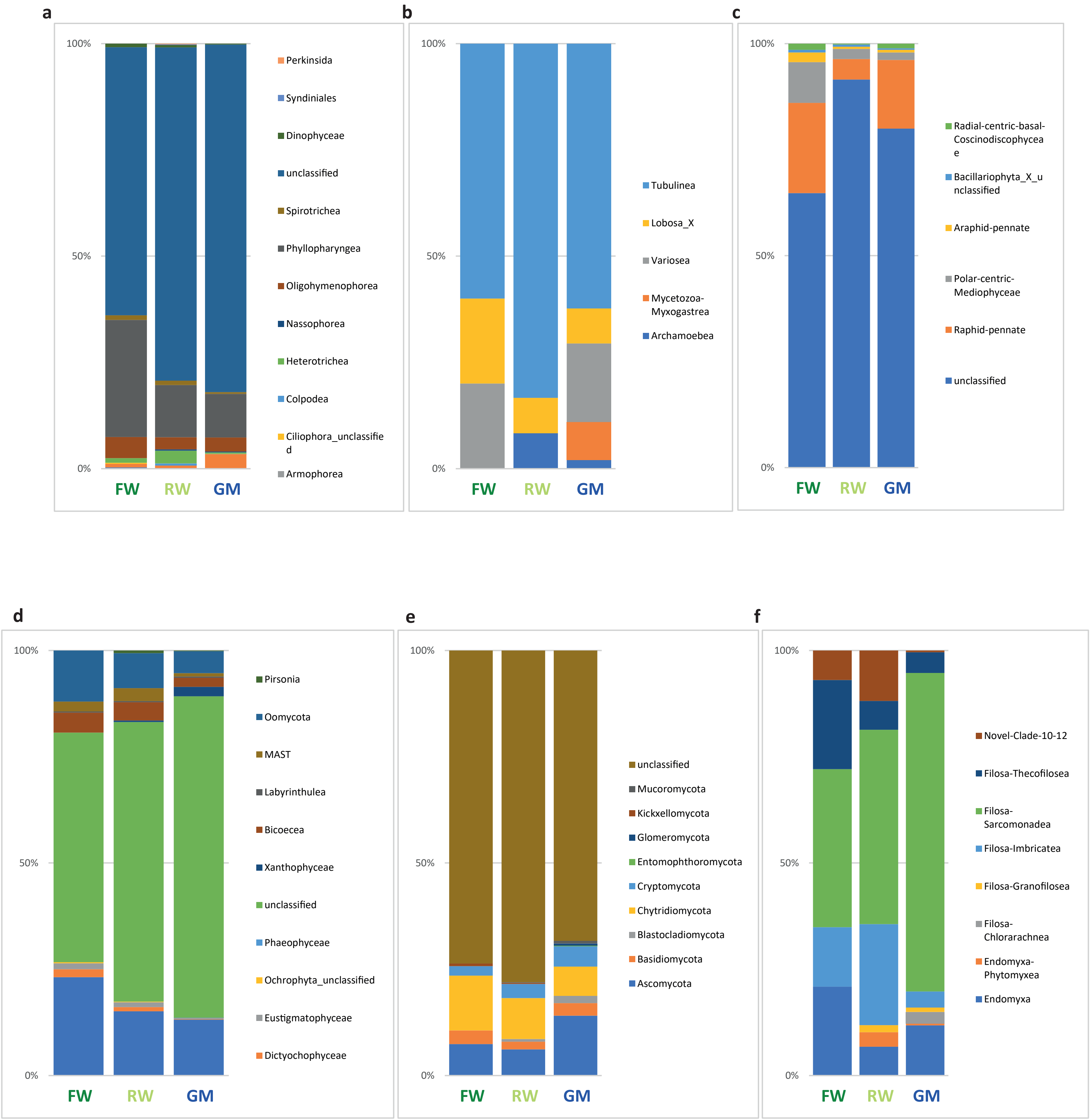

Supplementary Figure 2 | Taxonomic composition (in terms of relative abundance) of different groups in the three different compartments (FW, RW, GM). (**a**) Alveolata, (**b**) Amoebozoa, (**c**) Diatoms, (**d**) Other Stramenopiles, (**e**) Fungi, and (**f**) Rhizaria.
