# Supplementary material for: Aquatic urban ecology at the scale of a capital: community structure and interactions in street gutters
Source: ISME J. 2017 Oct 13;12(1):253–66. doi: 10.1038/ismej.2017.166 (PMC5739019; doi:10.1038/ismej.2017.166)
Supplement: Supplementary Figure 3 [file ismej2017166x8.pdf]

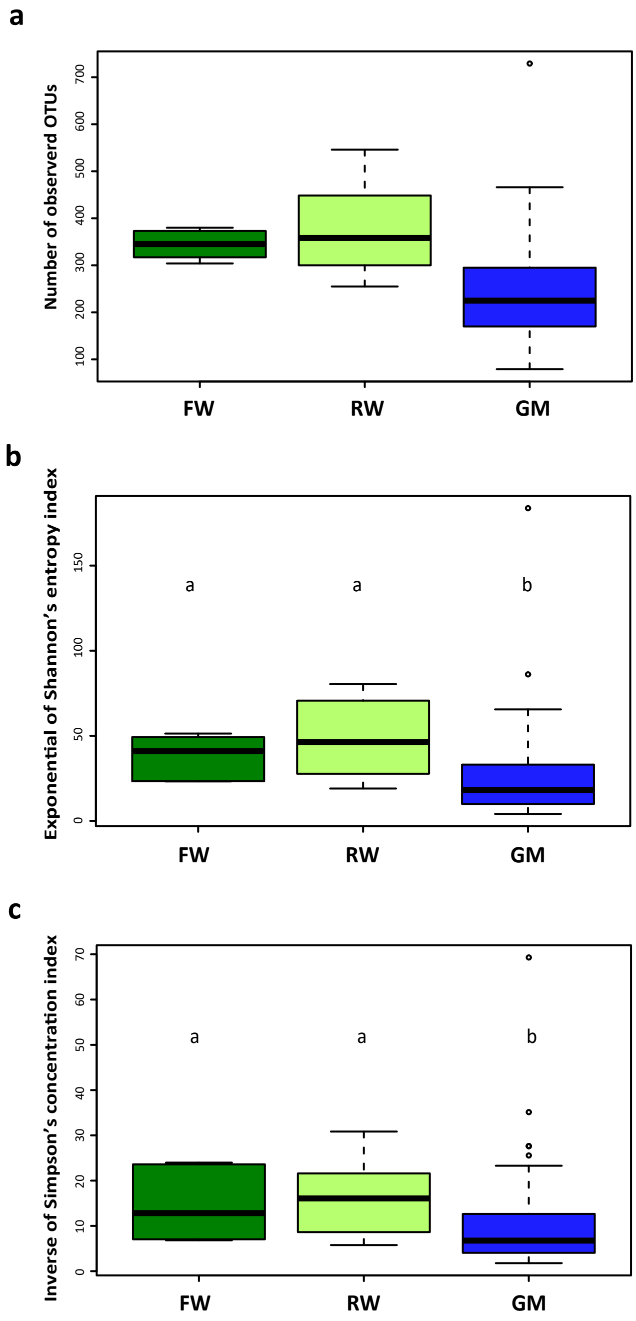

Supplementary Figure 3 | Measures of the alpha diversity in the three different compartments: flowing waters (FW, *n=6*), raw waters (RW, *n=8*), and gutter mats (GM, *n=90*). (**a**) First Hill number ^{0}D, *i.e.*, number of observed OTUs; (**b**) Second Hill number ^{1}D, *i.e.*, exponential of Shannon entropy index; (**c**) Third Hill number ^{2}D, *i.e.*, inverse Simpson concentration index. Small letters above the boxplots indicate significantly different distributions (Kruskal-Wallis, $p<0.05$).
