# Supplementary material for: Aquatic urban ecology at the scale of a capital: community structure and interactions in street gutters
Source: ISME J. 2017 Oct 13;12(1):253–66. doi: 10.1038/ismej.2017.166 (PMC5739019; doi:10.1038/ismej.2017.166)
Supplement: Supplementary Figure 4 [file ismej2017166x9.pdf]

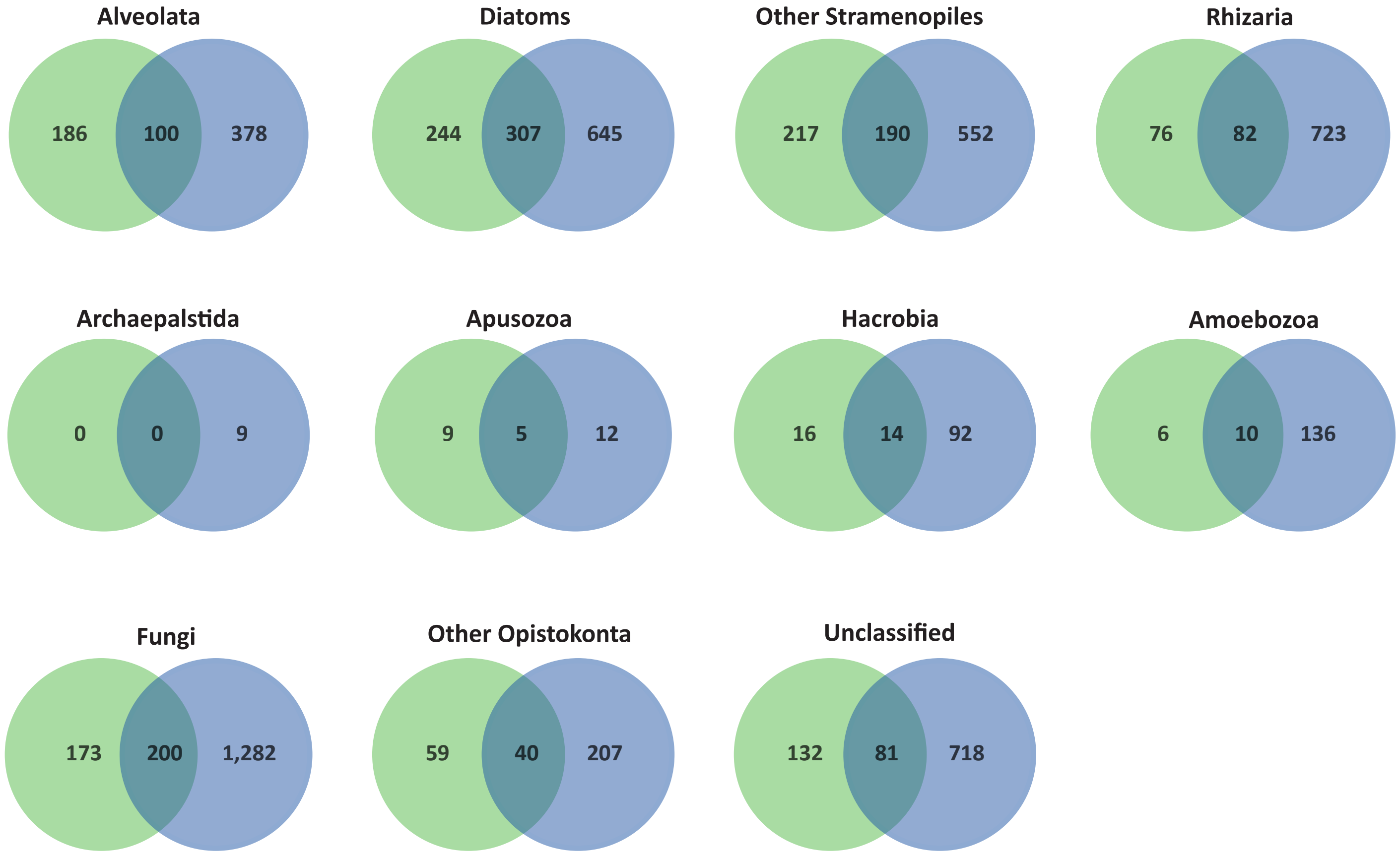

Supplementary Figure 4 | Venn diagrams representing the numbers of specific and shared OTUs among the non-potable water sources (NPS) and the gutter mats (GM) compartments for different taxonomic groups. The color codes correspond to gutter mats (*in blue*) and non-potable water sources (*in green*).
